# Self‐Immolative Gels: Programmable Degradation Using Self‐Immolative Linkers and Polymers

**DOI:** 10.1002/chem.70965

**Published:** 2026-04-04

**Authors:** Chuanfeng Li, Elizabeth R. Gillies

**Affiliations:** ^1^ Department of Chemistry The University of Western Ontario London Ontario Canada; ^2^ Department of Chemical and Biochemical Engineering The University of Western Ontario London Ontario Canada

**Keywords:** degradable, drug delivery, gels, hydrogels, polymers, self‐immolative

## Abstract

Gels have diverse structures and properties, making them of interest for a broad range of applications from pollution control to regenerative medicine. The controlled and stimulus‐triggered breakdown or property modulation of gels can lead to specific functions, such as sensing or drug release. Self‐immolative linkers (SILs) and self‐immolative polymers (SIPs) benefit from reaction cascades that allow a single stimulus‐mediated bond cleavage event to be translated across multiple bonds or even along an entire polymer chain. In this Concept article, we begin by introducing the chemistry fundamentals behind SILs and SIPs. Then, the incorporation of SILs into gel systems through cargo modification and their use in cross‐linkers is presented. We then discuss SIP organogels and how SIP systems have been imparted with the hydrophilic components needed to achieve hydrogels. A focus is given to the chemistry involved, and how this chemistry can be harnessed for potential applications. Finally, we present a summary and outlook for the field, examining the current status, and directions for future work.

## Graphical abstract

Self‐immolative linkers and polymers undergo bond‐cleaving reaction cascades in response to specific stimuli. Their incorporation into gels can be harnessed to release cargo, induce property changes, or trigger degradation in a highly controlled manner. These functions can be employed in applications such as sensing and drug delivery.

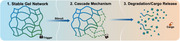

## 1 Introduction

Gels are three‐dimensional (3D) cross‐linked networks that can retain high mass fractions of liquid. If this liquid is water, they are typically referred to as hydrogels, while gels trapping organic liquids are termed organogels. Depending on their composition, gels can exhibit a wide range of properties, making them useful for many different applications. For example, organogels can be used as sorbents to remove oil spills [1], while hydrogels are commonly used in cosmetics, agriculture, and biomedical areas such as drug delivery and tissue engineering [2, 3, 4]. Increasingly, there is interest in controlling when and where hydrogels degrade [5]. In some cases, degradation may be desired at the end of product life to mitigate environmental pollution [6]. For in vivo applications, biodegradation is generally desired to enable clearance from the body [7, 8]. Stimuli‐responsive degradation can enable specific functions such as the targeted release of drugs in response to biological or external triggers [9], or the release of entrapped cells for cell sorting or tissue engineering [10, 11].

**FIGURE 1:**
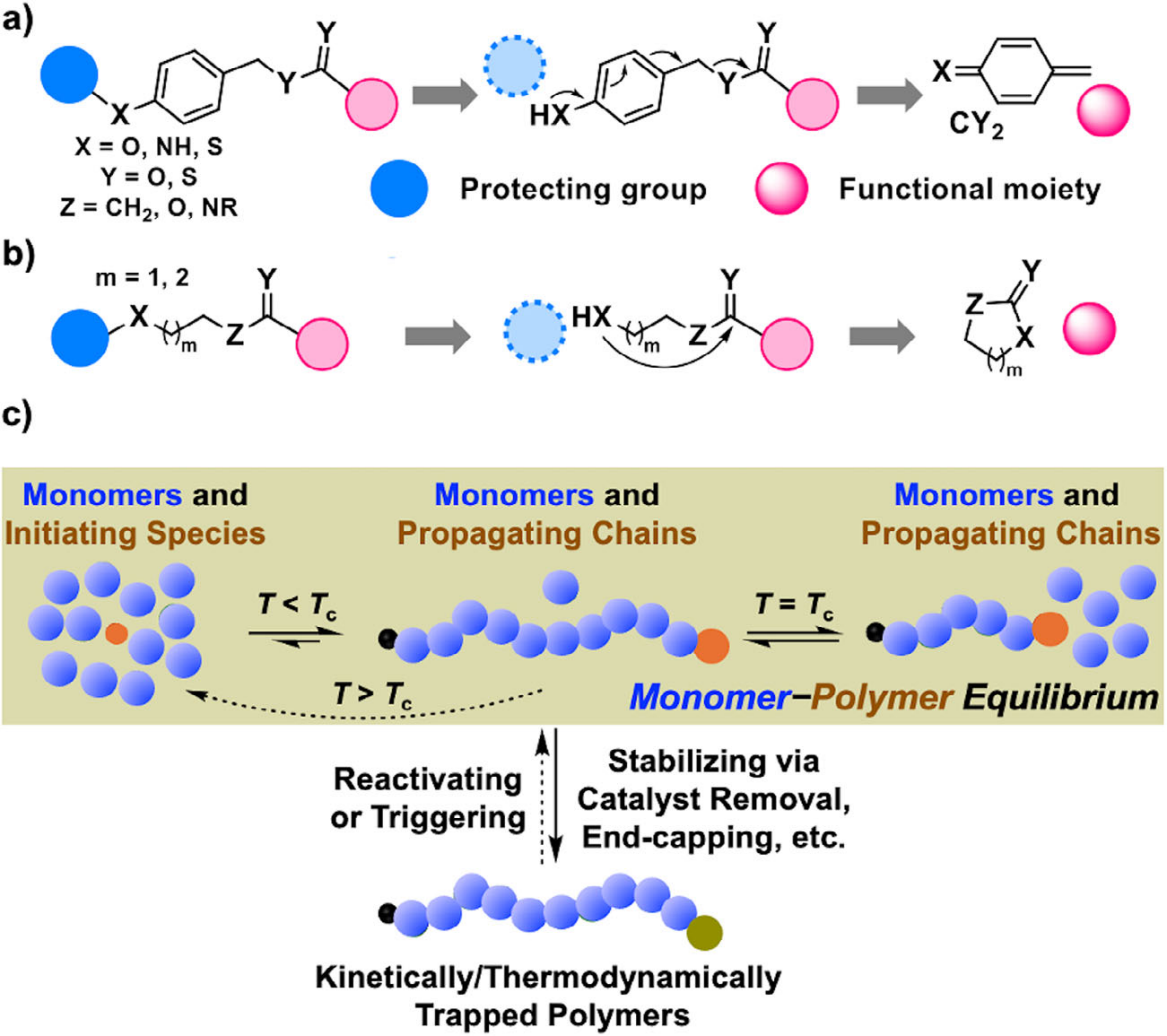
(a) Example SILs based on 1,6‐elimination reactions. (b) Example SILs based on intramolecular cyclization. (c) Schematic showing the monomer‐polymer equilibrium and chain capping for reversible stabilization of low *T_c_* SIPs. Adapted with permission from Ref. [57] (CC BY‐NC‐ND 4.0).

Self‐immolative polymers (SIPs) are a class of stimuli‐responsive degradable macromolecules under development over the past couple of decades [12, 13, 14]. They are characterized by their ability to translate a single stimulus‐mediated bond cleavage into a cascade of intramolecular reactions, resulting in molecular fragmentation. In general, there are two approaches to their design. In one approach, they are composed of self‐immolative linkers (SILs) [15, 16]. Such linkers were initially developed for prodrug chemistry and are capable of translating a bond cleavage at one terminus of the spacer to the other end of the spacer. Most examples involve either electron cascade mechanisms, such as 1,6‐eliminations (Figure 1a), or cyclization reactions to release 5‐ or 6‐membered rings (Figure 1b). These reactions can be combined along a polymer backbone such that end‐to‐end depolymerization of the SIP occurs upon backbone or end‐cap cleavage [17, 18]. In a second approach, SIPs can be composed of low ceiling temperature (*T_c_*) polymer backbones, which are stabilized by end‐capping (Figure 1c) [19, 20]. Upon removal of the end‐cap or backbone cleavage, depolymerization back to monomers occurs. One key advantage of SIPs is the ability to change the stimulus to which the polymer responds, by changing only the end‐cap, differentiating them from other stimuli‐responsive degradable polymers. Another advantage is their potential to amplify the initial bond cleavage through a cascade mechanism that can lead to changes in nanoscopic, microscopic, and macroscopic properties of materials.

**FIGURE 2:**
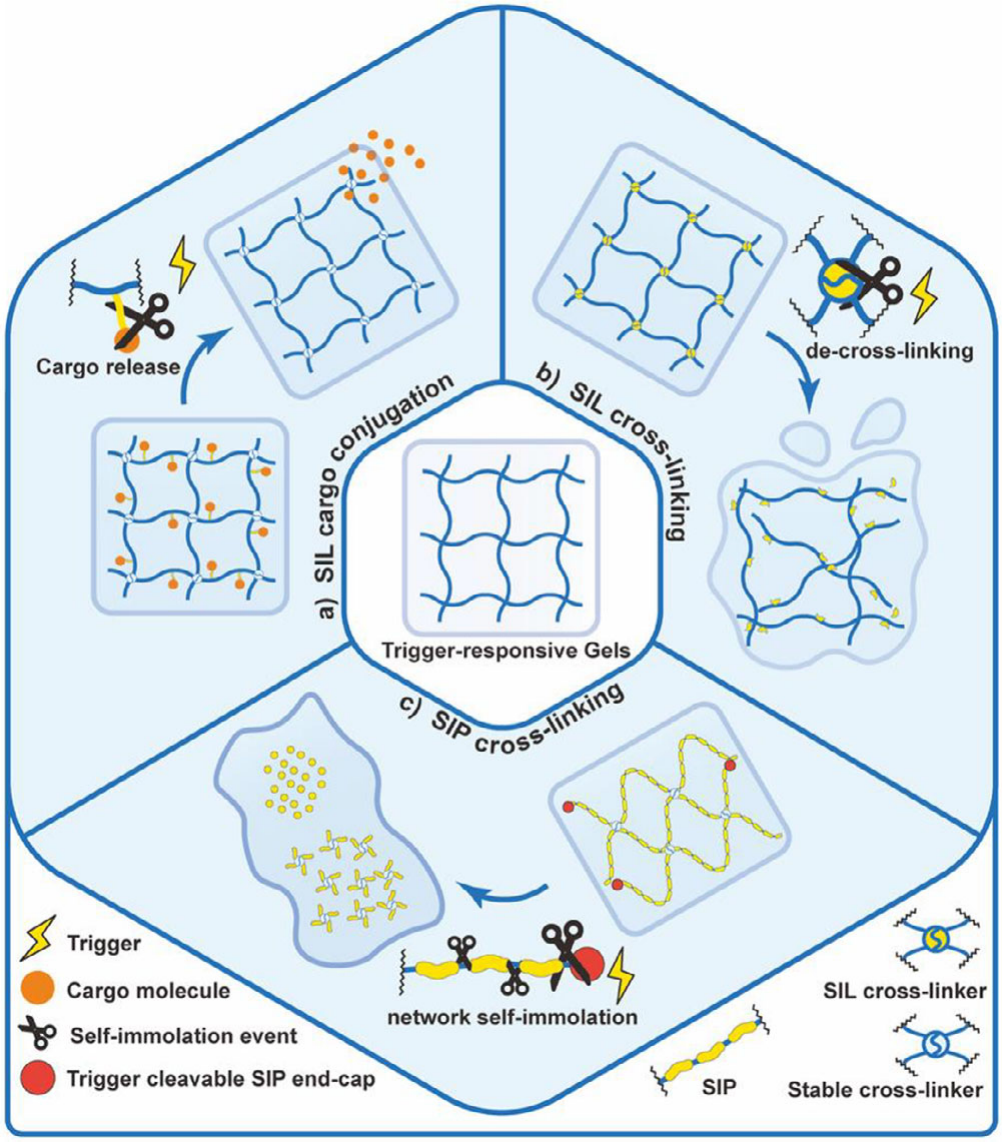
Example approaches to the incorporation of SILs and SIPs into gels: (a) chemical conjugation of cargo to the hydrogel by a SIL; (b) use of SILs within cross‐linkers; (c) cross‐linking of SIP backbones to form gels.

Gels can capitalize on the unique advantages of incorporating self‐immolative chemistry into a polymer network structure to achieve new functions and properties. In this Concept article, we will discuss the different approaches for incorporating SILs and SIPs into hydrogels. In the first section, we will present how SILs can be incorporated to either covalently attach (Figure 2a) or modify cargo molecules, leading to stimulus‐mediated cargo release or changes in hydrogel properties. This section will also discuss the use of SILs in hydrogel cross‐linkers, such that their cleavage leads to hydrogel degradation (Figure 2b). In the second section, we will discuss the incorporation of SIP backbones into organogels and hydrogels, such that triggered depolymerization leads to a chemically amplified breakdown of the gels (Figure 2c).

## 2 Gels Incorporating Self‐Immolative Linkers (SILs)

SILs enable trigger‐responsive disconnections, where an activation event can initiate a programmed cascade that breaks multiple covalent bonds. In gel systems, individual SILs can be incorporated through two different approaches. In one approach, they can be used to either covalently link or modify cargo molecules that are incorporated into gels cross‐linked by mechanisms such as free radical polymerization or non‐covalent interactions (e.g., hydrogen bonding). Stimulus‐mediated SIL cleavage results in the release of the drug or changes in hydrogel properties. In another approach, SILs can be used in the cross‐linking junctions that hold the network together, such that their stimulus‐mediated cleavage results in the breakdown of the network. In some cases, these approaches have been combined.

### 2.1 Self‐Immolative Linkages to Cargo

When SILs are used to modify hydrogel cargo, their cleavage can result in the release of the cargo or substantial changes in hydrogel properties. This approach does not formally involve amplification, as a single stimulus event typically leads to the release of one drug or partial changes in linker‐modified cargo. However, the advantage of this approach compared to conventional linker chemistry is that it enables the use of stimuli‐cleavable bonds that cannot be directly conjugated with functional groups on the cargo.

**FIGURE 3:**
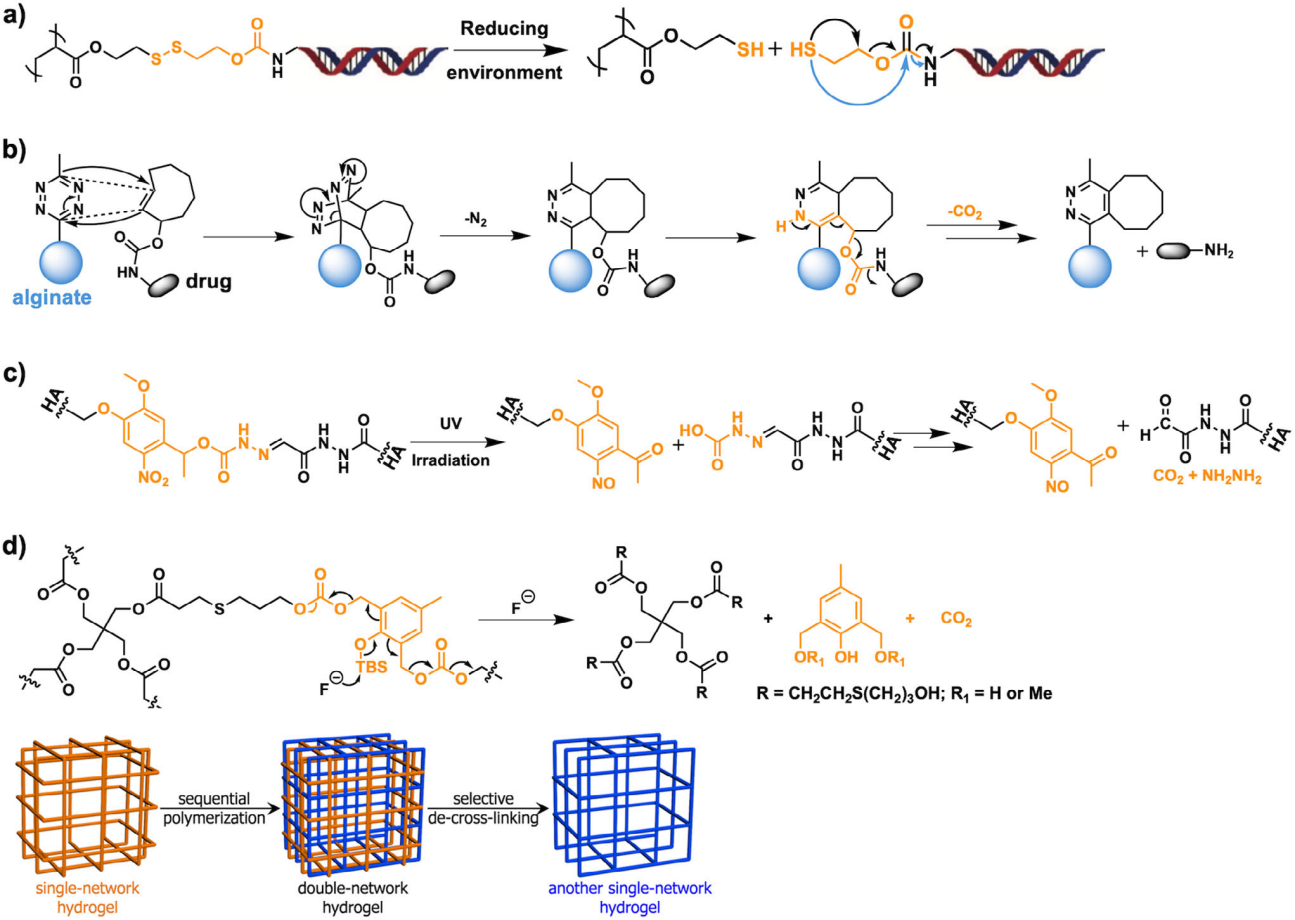
(a) Intramolecular thiol cyclization releases siRNA (two potential cyclization routes have been proposed). Adapted with permission from Ref. [22]. Copyright 2012, American Chemical Society. (b) Click to release approach where a Diels–Alder reaction between a prodrug and alginate hydrogel results in release of the active drug [27]. (c) Schematic showing the light‐degradable *o*‐nitrobenzyl and the hydrazone components of bifunctional linkers used to cross‐link HA [34]. (d) Schematic showing a SIL as part of the first network of a double network hydrogel. Fluoride triggers linker degradation and breakdown of the first network, leaving only the second network. Adapted with permission from Ref. [35]. Copyright 2018, American Chemical Society.

One common challenge with hydrogels is that encapsulated cargo tends to be released more rapidly than desired [21]. Covalent incorporation of drugs into hydrogel networks by SILs can provide controlled and stimuli‐responsive release. For example, Dunn et al. conjugated an amine‐terminated siRNA to a polymerizable acrylate group by a disulfide‐based self‐immolative linker (Figure 3a) [22]. This degradable macromer was then incorporated into nanogels via the particle replication in nonwetting templates technique. The biological reducing agent glutathione (GSH), which exists at elevated concentrations within cells and hypoxic tissues [23], was able to trigger disulfide cleavage, leading to an intramolecular cyclization that cleaved an adjacent carbamate linkage, thereby releasing the amine‐terminated siRNA. Some reports have suggested that the cyclization occurs to form a 5‐membered cyclic monothiocarbonate, while others have suggested the formation of a thiirane, followed by loss of CO_2_ [24, 25]. The nanogels were well tolerated by HeLa cells, although the cytotoxicity of the degradation products was not explicitly investigated. Using the same SIL chemistry, Ossipov et al. prepared hyaluronic acid (HA)‐based hydrogels with two different amine‐functionalized therapeutics conjugated: an oligonucleotide and pamidronate [26]. Nanogels and macroscopic hydrogels were formed by Ca^2+^ coordination of the pamidronate's phosphonate groups.

While not involving the direct conjugation of the drug to the hydrogel, Oneto et al. employed hydrogels with SIL chemistry based on the inverse‐electron demand Diels–Alder (IEDDA) reaction for the localized activation of small molecule prodrugs (Figure 3b) [27]. The approach involved an initial injection of an alginate hydrogel having pendent methyltetrazine groups near a tumor. Subsequently, a doxorubicin (Dox) prodrug with a *trans*‐cyclooctene (TCO) was injected. An IEDDA reaction of the TCO moiety with the hydrogel's methyl tetrazines activated a self‐immolative reaction cascade, resulting in the selective release of Dox near the tumor, thereby enhancing its therapeutic efficacy. Although the cytotoxicity of the Dox release byproducts was not investigated, Dox delivered via IEDDA activation resulted in lower systemic side effects compared to treatment with free Dox, indicating that the delivery system was well tolerated.

SILs were also used to incorporate single walled carbon nanotubes (SWNTs) into hydrogels. First, Yu et al. developed a conjugated poly(fluorene‐*co*‐phenylene) with pendent *tert*‐butyldimethylsilyl (TBS) ether protected 1,6‐elimination spacers, capable of coating the SWNT surface and thereby enabling high concentration dispersions of the nanotubes to be prepared, which would not otherwise be possible [28]. Upon addition of tetra‐*n*‐butylammonium fluoride (TBAF), the TBS group was cleaved, triggering a 1,6‐elimination reaction to remove the pendent groups, releasing more highly conductive SWNT materials as polymer pendent groups can reduce conductivity. In follow up work, these SIL‐functionalized polymer‐coated SWNTs were then combined with poly(vinyl alcohol) to form hydrogels via a freeze‐thaw cycling process [29]. Treatment with TBAF increased the hydrogel conductivity by removing the pendent groups. Furthermore, the resulting pendent hydroxy groups remaining on the polymer after linker fragmentation provided mechanical reinforcement of the hydrogels by contributing to hydrogen bond‐mediated cross‐linking.

In another example where SIL modification of hydrogel cargo was employed to modulate hydrogel properties, Kaufman et al. prepared and studied SIL‐modified thermo‐responsive elastin‐like polypeptides (ELPs) [30]. They site‐specifically conjugated reactive oxygen species‐cleavable boronic acid pinacol ester or β‐galactosidase‐cleavable galactose pendent groups using azide‐alkyne chemistry to unnatural 4‐propargyloxy‐L‐phenylalanine amino acids that were incorporated into the polypeptides. Stimulus‐mediated SIL cleavage resulted in large changes in lower critical solution temperature (LCST). These effects were then harnessed in hydrogels composed of physical mixtures of functionalized ELP and collagen that underwent physical gelation at 37°C. Treatment with H_2_O_2_ or β‐galactosidase substantially reduced the relative optical density and storage modulus (G’) of the hydrogels, showing potential for applications in drug delivery, sensing, and tissue engineering. However, as this SIL system would generate quinone methides, which can react with proteins [31], and are potentially cytotoxic [32], further studies will be needed.

### 2.2 Self‐Immolative Linkers as Cross‐Linkers

The use of SILs to cross‐link hydrogels enables the gels to be broken down in response to triggers to which the polymer backbone components are not inherently responsive. Again, this approach does not provide amplified breakdown of the network, but enables the incorporation of stimuli‐responsive groups onto polymer pendent groups that are not intrinsically responsive to the stimulus. For example, Wang et al. used a disuccinimidyl carbonate containing a disulfide bridge to cross‐link the amines on gelatin, stabilizing gelatin nanocarriers [33]. The cross‐linker also enabled the covalent conjugation of Dox's primary amine. In the presence of GSH at pH 5, mimicking the reducing and mildly acidic intracellular environment of tumor cells, reduction of the disulfide bond revealed thiols, which underwent intramolecular cyclization to de‐cross‐link the gelatin or release the Dox. The nanogels without Dox were well‐tolerated by a range of cell lines up to 200 µg/mL, although again the products of the self‐immolative reaction were not specifically studied. Ossipov et al. developed HA‐based hydrogels using heterobifunctional linkers with UV light‐cleavable moieties in the middle and terminal groups for either cross‐linking or drug conjugation [34]. For example, a linker having a pyridyl disulfide group on one terminus and a hydrazide group on the other terminus enabled cross‐linking of thiol‐functionalized HA with aldehyde‐functionalized HA (Figure 3c). Additional linkers having dopamine as a model drug at one terminus could also incorporated. Upon UV light irradiation, the *o*‐nitrobenzyl carbamate was cleaved, thus de‐cross‐linking the HA and/or releasing drug molecules. It should be noted that in this example, the self‐immolative nature of the linker was not technically used, as the work demonstrated only light‐mediated cleavage of the *o*‐nitrobenzyl group. However, cleavage of the disulfide under reducing conditions would be expected to lead to the same thiol cyclization chemistry as described above from Wang et al. [33].

Jung et al. developed double network hydrogels containing SILs (Figure 3d) [35]. The first network was prepared by cross‐linking a SIL composed of a TBS‐protected phenol having benzylic allyl carbonates in the *ortho* positions and a tetra‐thiol (pentaerythritol tetrakis(3‐mercaptopropionate); PETMP) cross‐linker via thiol‐ene click chemistry. Then, a second network was formed by free radical polymerization of *N*‐isopropylacrylamide and *N*,*N’*‐methylenebisacrylamide to form a double network hydrogel with tensile Young's modulus ninefold higher than that of the initial network. In the presence of fluoride as a stimulus, the TBS group was cleaved, resulting in 1,4‐elimination reactions to cleave the SIL, de‐cross‐linking the first network. The modulus of the hydrogel consequently decreased by 50‐fold and a reduction in LCST was observed from a broad transition ranging from 39°C to 53°C to a sharp LCST at 36°C. This macroscopic change was harnessed to demonstrate fluoride sensing in water through the release of colored dyes.

## 3 Gels Incorporating SIP Backbones

While self‐immolative linkers enable stimuli‐responsive drug release, changes in gel properties, and gel breakdown, their reactions remain chemically localized and unamplified. By incorporating SIP backbones into gels, a single stimulus‐mediated event can be translated into the complete depolymerization of a polymer backbone. This aspect is potentially advantageous in terms of achieving high stimulus sensitivity through true chemical amplification.

### 3.1 Self‐Immolative Organogels

**FIGURE 4:**
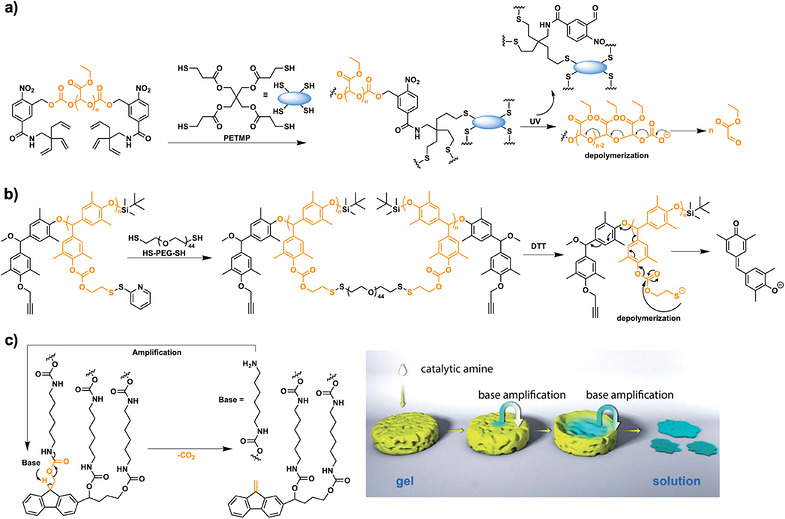
(a) Cross‐linking via a multifunctional end‐cap via thiol‐ene chemistry. Light irradiation leads to cleavage and PEtG depolymerization [36]. (b) Cross‐linking of a poly(benzyl ether) via pendent disulfide bond exchange. Depolymerization is initiated by DTT, translating the cleavage from the pendent group to the backbone [39]. (c) Catalytic amine base cleaves cross‐links in a polyurethane network, generating additional amines and thus an amplification cascade. Adapted with permission from Ref. [40] (CC BY‐NC 4.0).

As most SIP backbones were initially hydrophobic, and thus soluble in organic solvents, research began with the introduction of self‐immolative organogels. In one early example, Fan et al. prepared self‐immolative poly(ethyl glyoxylate) (PEtG) with UV light‐responsive *o*‐nitrobenzyl end‐caps functionalized with three alkenes at each terminus (Figure 4a) [36]. This polymer was then cross‐linked by thiol‐ene chemistry with PETMP in toluene using a free radical initiator. The resulting gel was depolymerized upon end‐cap cleavage by UV irradiation in 9:1 CD_3_CN:D_2_O. Another example involved the widely investigated self‐immolative polyphthalaldehyde (PPA) backbone. Soars et al. prepared PPA with pendent allyl ethers and end‐caps responsive to either light or fluoride [37]. This backbone is also inherently sensitive to strong acid. Organogels were prepared by thiol‐ene reactions with PETMP. They were rapidly degraded by treatment with acid or TBAF (fluoride sensitive gel). It was also found that the gels depolymerized upon sonication, presumably by mechanochemical cleavage, followed by depolymerization, as previously demonstrated for PPA [38] and PEtG [36].

In another early example, Xiao et al. prepared self‐immolative poly(benzyl ether)s with pendent pyridyl disulfides, enabling gel formation by reaction with bis‐mercapto‐terminated PEG to provide disulfide cross‐linking (Figure 4b) [39]. In this system, treatment with dithiothreitol (DTT) in THF resulted in cleavage of the disulfide, enabling cyclization and cleavage of the adjacent carbonate, followed by 1,6‐elimination to cleave the backbone and initiate depolymerization. Thus, the depolymerization was initiated from the pendent groups rather than the end‐cap and occurred unidirectionally, leaving partial chains for subsequent depolymerization. This approach has the advantage of increasing the concentration and availability of reactive triggers, but on the other hand it requires multiple triggering events to completely depolymerize the chains and thus the network.

Xu and coworkers explored a different approach to achieve amplification of the stimulus response in gels prepared from polymers with SILs in their backbones (Figure 4c) [40]. The polyurethane backbone contained base‐sensitive Fmoc derivatives that were cleaved in the presence of catalytic amines to generate further amines. Using a triol monomer, cross‐linked polyurethane networks were generated, which swelled in *N*‐methylpyrrolidone. Low concentrations of hexylamine induced gel degradation in an accelerating manner, consistent with an autocatalytic degradation process.

### 3.2 Self‐Immolative Hydrogels

Over the past few years, increasing efforts have been directed toward the preparation of self‐immolative hydrogels, through the incorporation of hydrophilic polymer blocks, cross‐linkers, and pendent groups to enable swelling in water. For example, Yin et al. found that PEtG‐PEG‐PEtG block copolymers underwent self‐assembly in water to form associative micellar gels [41]. The PEtG blocks formed the micelle cores and the PEG formed loops in the micellar corona as well as intermicellar bridges. The properties of the hydrogels were tuned based on the PEtG block lengths. However, the PEtG was not capped with stimuli‐responsive groups and depolymerization of the hydrogels was not investigated.

On the other hand, most examples have involved cross‐linking by covalent bonds. Building on the work of Fan et al. [36], Gong and coworkers prepared PEtG end‐capped with UV light‐responsive end‐caps bearing alkynes [42]. These polymers were cross‐linked with azide‐terminated 4‐arm PEG by Cu‐assisted azide‐alkyne cycloaddition chemistry (CuAAC) in 4/1 DMF/water. The organic solvent was necessary initially to dissolve the PEtG but the cross‐linked network could subsequently be transferred to and swollen in water. Degradation of the resulting hydrogels was triggered by light and was successfully turned on and off rapidly by starting and stopping the irradiation. This level of control was possible as end‐cap cleavage is the rate‐limiting step in the depolymerization process, with end‐to‐end depolymerization occurring rapidly thereafter. While the hydrophobic PEtG prevented gel formation in water, it provided domains in the hydrogel into which the hydrophobic drug celecoxib (CXB) was encapsulated. Light‐triggered depolymerization of the PEtG domains accelerated CXB release. However, even in the absence of irradiation, there was some background release of CXB. Molecules leached from the non‐loaded hydrogels exhibited moderate cytotoxicity at higher concentrations, but were non‐toxic at low concentration regardless of irradiation, suggesting no specific toxicity from the products of self‐immolation.

**FIGURE 5:**
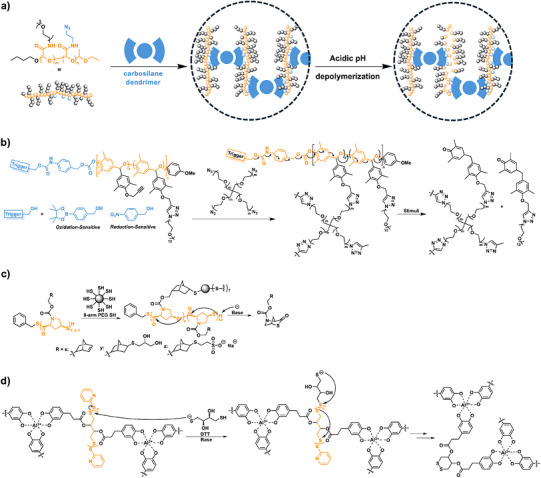
(a) PGAms with pendent hydrophilic groups and cross‐linkable azides are reacted with carbosilane dendrimers having peripheral alkynes by CuAAC to form hydrogels. Acidic pH cleaves the end‐cap, triggered PGAm depolymerization and network breakdown. Adapted with permission from Ref. [44]. Copyright 2025, American Chemical Society. (b) Cross‐linking and stimulus‐triggered depolymerization of a poly(benzyl ether) hydrogel where the stimulus can be changed by just switching the end‐cap [47]. (c) Cross‐linking and base‐induced depolymerization of a water‐soluble polythioester [48]. (d) Addition of DTT triggers depolymerization of a catechol‐functionalized polyDTT that is cross‐linked by complexation of Al^3+^.

Post‐polymerization, the pendent ethyl esters of PEtG can be reacted with amines forming polyglyoxylamides (PGAms) [43], thereby enabling the introduction of reactive cross‐linkable groups and tuning of the solubility and other properties for hydrogel formation. For example, starting from an acid‐responsive acetal end‐capped PEtG, Muñoz‐Sánchez et al. prepared PGAms with pendent hydrophilic tri(ethylene glycol) (TEG) chains as well as azides for cross‐linking (Figure 5a) [44]. Two different lengths of PGAm were reacted with carbosilane dendrimers having 4 or 8 peripheral alkynes by CuAAC in 9/1 THF/water and the resulting gels were transferred to water. This modular approach enabled tuning of the hydrogel properties such as swelling and elastic modulus. The hydrogels degraded at pH 5.5, resulting in a reduction in the modulus. Furthermore, curcumin was encapsulated, facilitated by the hydrophobic carbosilane dendrimer domains, and was released in an accelerated manner at mildly acidic pH.

As noted above, the physical encapsulation of drugs into hydrogels often results in relatively rapid release, even in the absence of a stimulus. To mitigate this background drug release, Gong et al. prepared an acetal end‐capped PGAm with pendent TEG and azide groups, and then used a portion of the pendent azides to introduce a UV light‐responsive linker that allowed covalent conjugation of amine‐containing drugs—in this case L‐phenylalanine methyl ester (PheMe) [45]. The remaining azides were reserved for cross‐linking with alkyne‐terminated 4‐arm PEG. This system still lacked sufficient water‐solubility for gelation in a fully aqueous system, but after cross‐linking in 4/1 DMF/water the gels could be transferred to water. Release of the amino acid model drug could be turned on or off based on irradiation, with no detectable background release in the absence of light. Depolymerization of the hydrogel occurred gradually at pH 6, triggered by acetal end‐cap cleavage, while the system was very stable at pH 7.4. No cytotoxicity was detected from the hydrogels, either with or without irradiation. This system shows promise for the stimulus‐triggered release of peptide‐based therapeutics, if the UV light trigger can be replaced with a stimulus more amenable to application in the human body.

Remaining issues with all of the above‐mentioned PEtG and PGAm hydrogel systems were their requirement for gelation in organic solvents and the use of potentially toxic copper species. This was addressed by Pardy et al. through the preparation of a PGAm with pendent 2‐hydroxyethyl amides, along with cross‐linkable azides [46]. This SIP was fully water‐soluble. Gelation was accomplished by strain‐promoted azide‐alkyne cycloaddition (SPAAC) using dibenzoazacyclooctyne‐terminated 4‐arm PEG. The formulation was amenable to cross‐linking upon mixing in a dual‐barrel syringe, making it promising for injectable formulations. The PGAm was equipped with an *o*‐nitrobenzyl ether end‐cap, enabling depolymerization in response to UV light. In future work, it would be ideal to replace the end‐cap with one responsive to an intrinsic biological stimulus. In addition, while drug loading and release were not yet investigated for this system, it would be beneficial to investigate covalent drug incorporation, particularly given the absence of hydrophobic domains for encapsulation of drugs by hydrophobic interactions.

Deng et al. prepared hydrogels based on a self‐immolative poly(benzyl ether) backbone (Figure 5b) [47]. Cross‐linkable alkynes and solubilizing PEG chains were incorporated as pendent groups, while a post‐polymerization end‐capping strategy was used to introduce end‐caps responsive to oxidizing or reducing stimuli. The polymers were cross‐linked with azide‐terminate 4‐arm PEG by CuAAC in THF and then transferred into water. Dox was loaded into the hydrogels and then selectively released upon application of the appropriate stimulus: H_2_O_2_ for the oxidation‐responsive gel and Na_2_S_2_O_4_ for the reduction‐responsive gel. This example highlights a key advantage of SIP hydrogels, which is that the stimulus to which the gel responds can be readily tuned by changing only the polymer end‐cap. However, this hydrogel would generate quinone methides upon depolymerization, which requires further assessment.

Sulfur‐containing SIP backbones have also been used for hydrogel formation. Soars et al. prepared norbornene‐functionalized self‐immolative 4‐hydroxyproline‐derived polythioesters and then functionalized them with 1‐thioglycerol and 2‐mercaptoethansulfonic acid to impart water‐solubility (Figure 5c) [48]. The remaining norbornene groups were then photocross‐linked with 8‐arm PEG thiol via thiol‐ene click chemistry to form hydrogels in water. The polythioesters were not end‐capped and consequently the hydrogels depolymerized in aqueous solution over time. The depolymerization was more rapid at pH 10 than at pH 7.4, which was attributed to deprotonation of the terminal thiol, leading to cyclization to release bicyclic monomer derivatives. Shen et al. prepared a penicillamine‐derived polythioester with pendent allyl groups and a disulfide end‐cap. Photocross‐linking in DMF by thiol‐ene chemistry led to gels that could partially swell when transferred to water (equilibrium water content = 60%). The poor swelling was attributed to hydrophobicity imparted by remaining allyl groups. The hydrogels could not be degraded in water, but degraded in DMSO upon addition of dithiothreitol indicating that if higher hydrophilicity and better swelling can be achieved, these hydrogels might be useful for releasing cargo in hypoxic environments such as tumors.

In another example highlighting a cross‐linking approach based on metal coordination, Agergaard et al. prepared a catechol‐functionalized self‐immolative polyDTT end‐capped with a pyridyl disulfide (Figure 5d) [49]. Cross‐linking was induced by adding aqueous NaOH to a MeOH solution of the polymer in the presence of Al^3+^, which deprotonated the catechol hydroxy groups, resulting in formation of tris‐catecholato‐Al^3+^ species, providing a cross‐linked network [49]. The gels were broken down by adding either DTT to induce depolymerization or 1 M HCl to break the catecholato‐metal cross‐links. Either process triggered the release of encapsulated rhodamine 6G dye as it resulted in breakdown of the hydrogel.

Finally, while all of the highlighted examples have involved linear SIPs, Gill et al. reported a hydrogel system based on self‐immolative dendrons [50]. The dendrons were composed of branched alternating cyclization and 1,4‐elimination spacers with peripheral alkynes and a light‐responsive *o*‐nitrobenzyl group at the focal point. The alkynes were cross‐linked with azide‐terminated 4‐arm PEG in 4/1 DMF/water, and then the gels were transferred to water. Upon UV irradiation, the *o*‐nitrobenzyl group was cleaved, initiating dendrimer fragmentation and a reduction in the compressive moduli of the hydrogels. The hydrogel prepared from the first generation dendron degraded more rapidly than that prepared from the second generation dendron, likely due to a lower level of cross‐linking and shorter molecular fragmentation pathway.

## 4 Summary and Outlook

Hydrogels are used in many different applications where stimuli‐responsive degradation at end of life, or for achieving specific functions such as sensing or drug release, can be beneficial. In this regard, self‐immolative chemistry can provide advantages beyond traditional stimuli‐responsive systems, such as high stimulus sensitivity through amplification and high platform modularity as linkers and end‐caps can be easily switched out to tune the stimulus. In this Concept article, we highlighted several different ways in which SILs and SIPs can be incorporated into hydrogel systems (Table 1). However, this chemistry is still in its relatively early stages. Thus far, a limited range of stimuli have been investigated, with most effort directed toward photochemically‐responsive and redox‐responsive end‐caps and linkers. In the future, it would be beneficial to focus on other biologically and environmentally relevant stimuli such as enzymes. In addition, systems responsive to applied stimuli such as ultrasound and near‐infrared light would enable a higher level of control over the degradation process by the hydrogel's user. It will also be important to study the potential toxicity of the SIL and SIP degradation products, due to their reactivity and potential to react with biomolecules. While a range of SIP backbones have been incorporated into hydrogels, these backbones are generally quite hydrophobic and require modification with hydrophilic pendent groups to prepare hydrogels. The backbones often provide hydrophobic domains that can aid in hydrophobic drug loading, but they also hinder access to solvent to facilitate depolymerization, making hydrogel degradation slower than desired in many cases. Therefore, it would be desirable to explore new SIP backbones that are inherently more hydrophilic. Furthermore, the scaled up synthesis of complex and multi‐functional gel systems will require careful consideration. Beyond networks prepared with SILs and SIPs used in polymeric systems, it is worth noting that SIL chemistry has also been incorporated into supramolecular hydrogel systems where triggered cleavage of small‐molecule gelators can lead to network degradation [51, 52, 53, 54]. Furthermore, other signal‐amplifying hydrogels have been developed using alternative unique chemical approaches [40, 55, 56]. Overall, we expect that the integration of self‐immolative and other chemical amplification approaches chemistry into gel network systems will continue to attract attention, with many interesting aspects to explore.

**TABLE 1:** Comparative summary of self‐immolative gels.

Classification	Self‐immolative linker/backbone	Degradation stimuli	Key points	References
SILs for cargo release (not amplified)	Disulfide with β‐carbamate.	GSH	Modified siRNA incorporated into a hydrogel prepared by free radical polymerization. Intramolecular cyclization of cleaved disulfide in hypoxic tissue results in release of siRNA.	Dunn [22]
	Disulfide with β‐carbamate.	GSH	Hyaluronic acid gel cross‐linked by Ca^2+^ coordination releases conjugated antisense oligonucleotides and a bisphosphonate drug.	Ossipov [26]
	Amino‐allyl carbamate generated upon IEDDA.	Bio‐orthogonal IEDDA reaction‐triggered cleavage	Injected tetrazine containing alginate hydrogel at tumor reacts with subsequently administered TCO prodrug of Dox to release the drug for selective anti‐tumor activity.	Oneto [27]
	tert‐Butyldimethylsilyl (TBS) ether protected 1,6‐elimination linker.	Fluoride	SIL‐protected polymer enables enhanced dispersion of SWNTs in a poly(vinyl alcohol) hydrogel and then cleavage reveals more conductive nanotubes.	Yu [29]
	Boronate or galactose‐protected 1,6‐elimination spacer.	Hydrogen peroxide (H_2_O_2_) or β‐ galactosidase (β‐gal)	Stimuli responsive phase transition of physical ELP‐collagen hydrogels with triggered release of hydrophobic model payload (Nile red).	Kaufman [30]
SILs as cross‐linkers (not amplified)	Disulfide with β‐carbamate.	GSH	The disuccinimidyl carbonate containing a disulfide bridge acts both as a cross‐linker and a Dox conjugation linker to disintegrate gelatin nanogels and release DOX upon cleavage.	Wang [33]
	*o‐*Nitrobenzyl carbamate, hydrazone, disulfide linkers.	UV light with potential for acid and GSH	UV light de‐cross‐links HA hydrogels and results in release of model drug dopamine.	Ossipov [34]
	TBS‐protected phenol having ortho benzylic allyl carbonates.	Fluoride	Double cross‐linked network prepared by thiol‐ene polymerization then free radical polymerization, where fluoride de‐cross‐links the first network changing the gel properties, for potential water sensing.	Jung [35]
SIL, with signal amplification capability	Polyurethane backbone modified with Fmoc derivatives.	Amine base	The polyurethane backbone contained base‐sensitive Fmoc derivatives that were cleaved in the presence of catalytic amines to generate further amines.	Xu [40]
SIP organogel (amplification)	PEtG with *o*‐nitrobenzyl carbonate end‐cap.	UV irradiation	Network is prepared by thiol‐ene polymerization and then light triggers network breakdown.	Fan [36]
	PPA with o‐nitrobenzyl carbonate or TBS end‐caps.	Light, fluoride, and mechanical cleavage	Network is prepared by thiol‐ene polymerization of PPA's pendent alkenes, then stimuli trigger network breakdown.	Soars [37]
	poly(benzyl ether) having pendent disulfides with β‐carbamate.	DTT	Network is prepared by disulfide exchange then DTT triggers breakdown from the pendent groups to the main backbone.	Xiao [39]
SIP hydrogel (amplification)	PEtG with *o*‐nitrobenzyl carbonate end‐cap.	UV irradiation	Network is prepared by azide‐alkyne cycloaddition, then light‐triggered depolymerization of the PEtG domains result in CXB release.	Gong [45]
	PGAms with pH‐responsive end‐cap and *o*‐nitrobenzyl carbamate linkage to model drug.	UV irradiation and acidic pH	Network is prepared by azide‐alkyne cycloaddition. A drug conjugated to slow background release but can be triggered in an on–off manner by light. Mildly acidic pH triggers network breakdown.	Gong [42]
	PGAms with pH‐responsive end‐caps.	Acidic pH	Network is prepared by azide‐alkyne cycloaddition with a carbosilane dendrimer. A hydrogel with encapsulated curcumin was degraded at mildly acidic pH with release of cargo.	Munoz [44]
	Poly(benzyl ether)s with either boronate or 4‐nitrobenzyl carbamate end‐caps.	Oxidizing or reducing stimuli	Modular post‐polymerization end‐capping allows different stimuli‐responsive end‐caps to be easily introduced then cross‐linking performed by azide‐alkyne cycloaddition. Dox can be encapsulated and released with oxidizing or reducing stimuli.	Deng [47]
	Polythioesters with thiol terminus.	Basic pH	Network is prepared by thiol‐ene chemistry. Hydrogels depolymerize more rapidly at pH 10 than at pH 7.4.	Soars [48]
	PolyDTT with pendent metal‐chelating catechols and terminal disulfides.	DTT or acidic pH	Network is prepared by metal binding to catechols. DTT triggers backbone depolymerization while acid cleaves metal coordination cross‐links. Encapsulated rhodamine 6G dye is released upon network breakdown.	Agergaard [49]
	Dendron with branched alternating cyclization and 1,4‐elimination spacers and an *o*‐nitrobenzyl carbamate focal point.	UV irradiation	Network is prepared by azide‐alkyne cycloaddition. Light triggers dendrimer fragmentation and network breakdown, resulting in a reduction in compressive moduli.	Gill [50]
			

## Conflicts of Interest

The authors declare no conflicts of interest.

## Data Availability

Data sharing not applicable to this article as no datasets were generated or analyzed during the current study.
